# Respiratory syncytial virus hospitalization risk in the second year of life by specific congenital heart disease diagnoses

**DOI:** 10.1371/journal.pone.0172512

**Published:** 2017-03-02

**Authors:** Deborah Friedman, Jon Fryzek, Xiaohui Jiang, Adam Bloomfield, Christopher S. Ambrose, Pierre C. Wong

**Affiliations:** 1 Department of Pediatrics, New York Medical College, Valhalla, NY, United States of America; 2 EpidStat Institute, Rockville, MD, United States of America; 3 AstraZeneca, Gaithersburg, MD, United States of America; 4 Division of Cardiology, Children's Hospital, Los Angeles, CA, United States of America; National Yang-Ming University, TAIWAN

## Abstract

Children with hemodynamically significant congenital heart disease (CHD) are at elevated risk of morbidity and mortality due to respiratory syncytial virus (RSV) disease compared to their healthy peers. Previous studies have demonstrated lower RSV hospitalization risk among all children with CHD at 12–23 months of age versus 0–11 months of age. However, RSV hospitalization risk at 12–23 months of age by specific CHD diagnosis has not been characterized. Both case-control and cohort studies were conducted using data from the US National Inpatient Sample from 1997 to 2013 to characterize relative risk of RSV hospitalization among children 12–23 months of age with CHD. Related CHD diagnoses were combined for analysis. Hospitalizations for RSV and unspecified bronchiolitis were described by length of stay, mechanical ventilation use, mortality, and total charges. Over the 17-year period, 1,168,886 live birth hospitalizations with CHD were identified. Multiple specific CHD conditions had an elevated odds ratio or relative risk of RSV hospitalization. Mean total RSV hospitalization charges were significantly higher among children with CHD relative to those without CHD ($19,650 vs $7,939 in 2015 dollars) for this period. Compared to children without CHD, children with Ebstein’s anomaly, transposition of the great arteries, aortic stenosis, heterotaxia, and aortic arch anomalies had 367-, 344-, 203-, 117- and 47-fold increased risk of inpatient RSV mortality, respectively. Unspecified bronchiolitis hospitalization odds and relative risk across CHD diagnoses were similar to those observed with RSV hospitalization; however, unspecified bronchiolitis hospitalizations were associated with shorter mean days of stay and less frequently associated with mechanical ventilation or mortality. Among children with more severe CHD diagnoses, RSV disease remains an important health risk through the second year of life. These data can help inform decisions regarding interventions to protect children with CHD from severe RSV disease during their second year of life.

## Introduction

Respiratory syncytial virus (RSV) is a common pediatric respiratory infection, with nearly all children affected by 2 years of age [1]. More than 170,000 children under 5 years of age are hospitalized annually in the United States (US) due to RSV, with hospitalization rates highest among children less than 1 year of age [2, 3]. Morbidity and mortality associated with RSV are increased among certain populations, including children with hemodynamically significant congenital heart disease [4–6]. When hospitalized, children with hemodynamically significant CHD can experience a more complicated clinical course, with a high proportion requiring intensive care unit admission, assisted ventilation, longer duration of oxygen supplementation, and prolonged duration of hospital stay [7–10]. In addition, congenital heart disease (CHD) has been associated with a 3.7-fold increased risk of death among infants hospitalized for RSV [11].

Immunoprophylaxis with palivizumab has demonstrated reduced RSV-related hospitalization in high-risk groups, including preterm infants ≤35 weeks’ gestational age and children ≤24 months of age with bronchopulmonary dysplasia or hemodynamically significant CHD [12, 13]. In a study of 1287 children ≤24 months of age with hemodynamically significant CHD, palivizumab was associated with a 45% relative reduction in RSV hospitalizations compared with placebo (*P* = .003), a 56% reduction in days of RSV hospitalization per 100 children (*P* = .003), and a 73% reduction in total RSV hospitalization days with increased supplemental oxygen per 100 children (*P* = .014) [13]. A recent single-center case-control study demonstrated a 75% reduction (*P* < .001) in lower respiratory tract infection (LRTI)-related hospitalization, 65% reduction (*P* = .008) in LRTI-related intensive care unit admissions, and 49% reduction (*P* = .02) in LRTI-related mortality in infants with CHD who received palivizumab prophylaxis versus those who did not [14].

In 2003, the American Academy of Pediatrics (AAP) released a policy statement recommending RSV immunoprophylaxis in children ≤24 months of age with hemodynamically significant cyanotic and acyanotic CHD [15]. The guidelines noted that children with CHD who were most likely to benefit from immunoprophylaxis included those <12 months of age with cyanotic heart disease, those receiving medication to control congestive heart failure, or those with moderate-to-severe pulmonary hypertension. In 2014, the AAP guidance was updated to recommend against RSV immunoprophylaxis in children with CHD in the second year of life (ie, age 12–23 months at the start of the RSV season), asserting that these children are not at increased risk of severe RSV infection [16]. In support of this decision, the Committee on Infectious Diseases (COID) and Bronchiolitis Guidelines Committee policy statement cited an analysis of Tennessee Medicaid data for children younger than 3 years, conducted from July 1989 to June 1993, that reported RSV hospitalization rates among children with any form of CHD as 120.0 per 1000 children for children 0 to <6 months of age, 63.5 per 1000 children for children 6 to <12 months of age, and 18.2 per 1000 children for children 12 to <24 months of age. The RSV hospitalization rate for children with CHD at 12–23 months of age was less than half the hospitalization rate for low-risk infants in the first 5 months after birth (44.1 per 1000) [6, 16]. However, although all children with CHD have a lower risk of RSV hospitalization in their second year of life, given the heterogeneity of CHD, it remains unclear whether specific CHD diagnoses are associated with higher risk of hospitalization due to RSV during the second year of life. This study was designed to investigate the risk of hospitalization due to RSV in the second year of life by specific CHD diagnostic groups using a large, nationally representative database of US inpatient admissions.

## Materials and methods

### Study design

Cohort and case-control studies were conducted using data from the 1997–2013 National Inpatient Sample database to estimate RSV hospitalization rates at 12–23 months of age among children diagnosed with various types of CHD [17]. The case-control approach was considered the primary analysis for the estimate of the incidence of RSV hospitalization at 12–23 months of age, owing to the recognized limitations of the cohort approach as detailed below. The NIS is a 20% stratified sample of US inpatient admissions to US acute care hospitals and has been previously used to examine national trends in health risks related to CHD [18]. Both clinical and non-clinical data elements for each hospital stay are available in the NIS, including patient demographics, primary and secondary diagnoses and procedures, resource-use information, and severity and comorbidity measures. National estimates can be calculated using weighting provided by the Agency for Healthcare Research and Quality. The National Inpatient Sample database is a publically available database, in which patient records are anonymized and de-identified before public release. Analyses of de-identified data from the National Inpatient Sample database are exempt from federal regulations for the protection of human research participants. All procedures involving human participants and confidentiality were reviewed and approved by the Research Ethics Review Board of the Agency for Healthcare Research and Quality.

### Patient identification

Congenital heart defects were identified by the study physicians’ review of available ICD-9 codes and informed by the technical specifications of the pediatric heart surgery volume quality indicator published by the Agency for Healthcare Research and Quality [19]. Because many CHD diagnoses are extremely rare, the authors grouped defects that commonly occur together, are difficult to distinguish from each other, and/or produce the same circulatory effect (**Table 1**). Consistent with established standards in CHD surveillance [20], infants with a code for preterm birth (ICD-9 765.0x, 765.1x, 765.20–765.28) or birthweight <2000 grams (764.0x, 764.1x, 764.2x, and 764.9x with 5th digit from 0 to 7, or V2130, V2131, V2132, V2133, V2134) were excluded from the birth cohort of infants with atrial septal defect or patent ductus arteriosus. As noted in **Table 1**, five diagnostic groups were analyzed in the absence of other CHD diagnoses due to a high coincidence with other CHD diagnoses.

**Table 1:** ICD-9-CM^a^ Codes for Conditions Associated With Congenital Heart Disease.

Conditions	ICD-9-CM	Average Annual Live Births 1997–2013
Isolated patent ductus arteriosus^b^^,^^c^	747.0	8184
Unspecified congenital anomaly of heart, other specified congenital anomaly of heart	746.89, 746.9	7770
Isolated unspecified defect of septal closure, ventricular septal defect^b^	745.4, 745.9	7169
Isolated secundum atrial septal defect, partial anomalous pulmonary venous return^b^^,^^c^	745.5, 747.42	5544
Isolated pulmonary valve stenosis, congenital anomalies of pulmonary artery, other congenital anomalies of pulmonary valve, congenital pulmonary valve anomaly unspecified^b^	746.00, 746.02, 746.09, 747.3	3286
Pulmonary valve atresia, tricuspid atresia, pulmonary artery coarctation and atresia, other anomalies of pulmonary artery and pulmonary circulation	746.01, 746.1, 747.31, 747.39	1331
Congenital anomalies of aortic arch with other congenital anomalies of aorta, coarctation, interrupted aortic arch, congenital anomaly of aorta unspecified	747.10, 747.11, 747.20, 747.21, 747.29	1231
Tetralogy of Fallot, infundibular pulmonic stenosis	745.2, 746.83	1088
Congenital mitral stenosis, congenital mitral regurgitation	746.5, 746.6	895
Congenital insufficiency of aortic valve	746.4	742
Other endocardial cushion defect, ostium primum defect, unspecified	745.60, 745.61, 745.69	659
Subaortic stenosis, congenital atresia and stenosis of aorta, congenital aortic stenosis, congenital obstructive anomalies	746.3, 746.81, 746.84, 747.22	638
Congestive heart failure	428.0	537
Transposition of great vessels	745.10, 745.12 745.19	534
Hypoplastic left heart syndrome	746.7	523
Malposition of heart and cardiac apex with other anomalies of great vessels (heterotaxia), congenital anomalies of great veins	746.87, 747.40, 747.49	513
Double outlet right ventricle, other bulbus cordis anomalies	745.11, 745.8	387
Common truncus	745.0	169
Ebstein's anomaly	746.2	164
Cardiomyopathy	425.4	162
Congenital heart block	746.86	132
Common ventricle, cor biloculare	745.3, 745.7	125
Total anomalous pulmonary venous connection	747.41	109
Isolated coronary artery anomaly congenital^b^	746.85	37
Cor triatriatum	746.82	14

^a^ ICD-9-CM, International Classification of Diseases, Ninth Revision, Clinical Modification.

^b^ Due to a high coincidence with other CHD diagnoses, these diagnostic groups were analyzed in the absence of other CHD diagnoses.

^c^ Excludes preterm births with atrial septal defect or patent ductus arteriosus.

### Outcome measures and statistics

Patient demographics were described using summary statistics. Any hospitalization with at least one of the three RSV-specific ICD-9 codes (ICD-9 480.1, 466.11, and 079.6) was categorized as a hospitalization due to RSV. Although laboratory confirmation is unknown for cases coded as RSV, a previous study demonstrated that these codes are specific for diseases related to RSV [21]. As the most common disease associated with RSV in young children is bronchiolitis, hospitalizations coded as unspecified bronchiolitis (UB) were also evaluated as a comparison outcome. For this analysis, UB hospitalizations were those with a diagnosis code of 466.19 (acute bronchiolitis due to other infectious organisms) or 466.1 (acute bronchiolitis) without any RSV-specific diagnostic code. Although UB-coded hospitalizations can include events due to undiagnosed RSV, UB-coded events commonly represent bronchiolitis caused by pathogens other than RSV [21].

Hospitalizations for RSV and UB were described by length of stay, use of mechanical ventilation, inpatient mortality, and total charges. Inpatient mortality (coded as “Died” in the National Inpatient Sample dataset) and mechanical ventilation use (procedure codes 9390, 9392, 9601, 9602, 9603, 9604, 9605, 9670, 9671, or 9672) were evaluated as a proportion of total hospitalizations. Because length of hospitalization and total hospital charges were not normally distributed, geometric means were calculated. Total charges were inflation-adjusted to 2015 dollars [22].

For the cohort analysis, hospitalization rates were calculated using the weighted estimate of hospitalizations for children 12–23 months of age and estimates of all infant birth hospitalization discharges with CHD diagnoses for 1997–2013, excluding neonatal deaths. As the absolute risks of RSV hospitalization are not robust due to limited testing and coding for RSV [23–25], the relative risks (RR) of hospitalization for UB were calculated for children 12–23 months of age with CHD diagnoses relative to those 12–23 months of age without CHD.

However, there are limitations with using a cohort design with birth hospitalization discharge diagnoses as the denominator for RR calculations, as some forms of CHD are frequently diagnosed only after the birth hospitalization and some may be diagnosed inaccurately at birth [20]. CHD codes may not be assigned to all children with a past history of the condition during hospitalizations in the second year of life, particularly for CHD diagnoses that have become clinically insignificant due to surgical intervention or clinical resolution, and no data were available on mortality among children with CHD following the birth hospitalization. As a result, a case-control analysis was also conducted using data exclusively collected for children hospitalized at 12–23 months of age.

For the case-control analysis, for children with and without CHD, the odds of being hospitalized with RSV or UB were compared to the odds of having a hospitalization event expected to have a similar incidence in children with and without CHD. ICD-9 codes for traumatic injuries, burns, and poisonings (ICD-9 codes 800–999) were used as the comparison outcome, excluding diagnoses that were found to be disproportionately more frequent among children with CHD, specifically those related to surgical procedures or unspecified causes (ICD-9 codes 990–999), foreign bodies by mouth (ICD-9 codes 933–935), and femoral neck fractures (ICD-9 code 820). Given the frequency of RSV hospitalization, the odds ratio (OR) should approximate the true RR among children diagnosed with CHD at 12–23 months of age. Because of the known limitations of the cohort approach, the case-control approach was considered the primary analysis for the estimate of the incidence of RSV hospitalization at 12–23 months of age.

To examine temporal trends in the results, and given the small sample for many CHD diagnostic groups, the analysis was conducted separately and compared for 1997–2005 and 2006–2013, based on dividing the total surveillance period at the mid-point. All data management and analyses for this study were performed using SAS/STAT software, version 9.3 of the SAS System (SAS Institute Inc., Cary, NC, USA), with statistical procedures that incorporated weights to account for the structure of the sample survey data. Statistical significance was assigned for a *P* value < .05.

## Results

Over the 17-year period studied (1997–2013), a total of 1,168,886 live birth hospitalizations with CHD ICD-9 codes were identified (annual average of 68,758 births). As expected, the prevalence of CHD diagnoses varied considerably by diagnostic group, with patent ductus arteriosus, unspecified anomaly, atrial septal defect, and ventricular septal defect representing the most prevalent diagnoses (**Table 1**). The coincidence of CHD diagnostic groups is summarized in S1 Table (live births) and S2 Table (children 12–23 months). The surveillance period included 4,813 RSV hospitalizations among children 12–23 months of age with CHD. The proportion of children with non-private insurance was higher among those with RSV hospitalization at 12–23 months of age for children with and without CHD (both *P* < .01) compared to the proportion among the live birth populations (**Table 2**). The proportion with Native American race (*P* < .01) was also higher among RSV-hospitalized infants compared to the birth population; whereas the proportion with White race (*P* = .04) was lower. The timing of RSV hospitalization matched the known seasonality of RSV in the US [26].

**Table 2:** Characteristics of Hospitalized Patients 12–23 Months of Age in the NIS, 1997 to 2013.

		With Any CHD			Without CHD		
		Live Births	All Age 12–23 Months Hospitalizations	Age 12–23 Months RSVH	Live Births	All Age 12–23 Months Hospitalizations	Age 12–23 Months RSVH
Total hospitalizations, n		1,168,886	99,621	4,813	65,333,543	3,085,545	224,044
Sex, %						*	*
	Male	51.4	51.7	51.6	51.1	56.2	55.7
	Female	48.6	48.3	48.4	48.8	43.7	44.3
Race or ethnic group, %			*	*		*	*
	White	38.6	40.3	34.3	41.7	38.9	40.1
	Black	15.0	12.2	15.5	10.4	14.5	12.7
	Hispanic	20.0	16.5	20.2	16.9	15.5	15.4
	Asian or Pacific Islander	3.3	3.4	2.7	3.7	2.4	2.2
	Native American	0.6	0.9	1.4	0.6	0.8	1.5
	Other	4.3	4.4	3.5	4.2	4.1	3.8
Admission quarter, %			*	*		*	*
	January–March	21.6	25.7	58.4	22.4	31.7	62.1
	April–June	22.3	23.4	7.6	23.1	20.5	5.6
	July–September	23.2	20.6	2.1	24.6	16.0	1.5
	October–December	22.3	21.3	22.9	23.1	22.2	23.6
Type of health insurance, %			*	*		*	*
	Private	48.7	40.6	35.7	52.1	43.1	39.4
	Other	51.1	59.2	64.2	47.7	56.6	60.3

RSVH, Respiratory Syncytial Virus Hospitalization.

*P < .05 compared to the proportion among live births.

Among CHD patients hospitalized for RSV disease at 12–23 months of age, the mean length of stay was 4.5 days; 12% required mechanical ventilation, and 1.5% died (**Table 3**). Length of stay and mechanical ventilation use varied considerably across diagnostic groups and were highest among those with cardiomyopathy, congestive heart failure, and transposition of the great vessels. Mean total hospitalization charges ($19,650) were significantly higher than those observed in children without CHD ($7,939) (*P* < .01) and were higher in those conditions with increased mean hospital lengths of stay. Inpatient mortality was highest among children with transposition of the great vessels (10.2%), congestive heart failure (9.2%), cardiomyopathy (9.2%), Ebstein’s anomaly (8.8%), and aortic stenosis (6.1%) (**Table 4**). Compared to children 12–23 months of age without CHD, children with Ebstein’s anomaly, transposition of the great arteries, aortic stenosis, heterotaxia, and aortic arch anomalies had 367-fold, 344-fold, 203-fold, 117-fold, and 47-fold increased risk of RSV mortality, respectively (**Table 4**). Among those with inpatient mortality, 87% required mechanical ventilation. For UB, mean hospital lengths of stay were shorter and hospitalization costs were lower than for RSV hospitalization, and mechanical ventilation use was less prevalent (**Table 5**). Inpatient mortality was rare and significantly less frequent than with RSV; the only mortality events observed occurred among children with heterotaxia or congestive heart failure (**Table 4**).

**Table 3:** RSV Hospitalization Illness Severity Indicators for Children 12–23 Months of Age With and Without CHD, 1997–2013.

Condition^a^	12–23 Months RSVH, N	Mean^b^ Days of Stay (SE)	Percent of Mechanical Ventilation Use (SE)	Mean^b^^,^^c^ Total $ Charge (SE)
Transposition of great vessels	138	4.6 (1.3)*	30.7 (7.6) *	34485 (10757) *
Congestive heart failure	356	8.5 (1.4) *	33.9 (5.5) *	45631 (7702) *
Cardiomyopathy	156	6.2 (2.0) *	29.3 (9.2) *	34020 (9960) *
Ebstein's anomaly	52	3.4 (0.6) *	8.8 (8.4)	15198 (4964) *
Subaortic stenosis, congenital atresia and stenosis of aorta, congenital aortic stenosis, congenital obstructive anomalies	163	3.5 (0.4) *	6.1 (4.2)	13573 (2235) *
Malposition of heart and cardiac apex with other anomalies of great vessels (heterotaxia), congenital anomalies of great veins	149	4.8 (1.7) *	19.7 (8.2) *	33365 (8618) *
Congenital anomalies of aortic arch with other congenital anomalies of aorta, coarctation, interrupted aortic arch, congenital anomaly of aorta unspecified	172	4.3 (0.8) *	16.8 (6.3) *	21082 (4576) *
Isolated patent ductus arteriosus	212	4.8 (0.7) *	13.8 (5.3) *	18921 (3445) *
Isolated secundum atrial septal defect, partial anomalous pulmonary venous return	801	5.3 (0.4) *	15.1 (2.6) *	22502 (2375) *
Common ventricle, cor biloculare	63	6.6 (2.3) *	22.1 (12.4) *	31810 (15718) *
Total anomalous pulmonary venous connection	43	4.1 (1.6)	21.8 (18.2) *	24579 (15049)
Congenital insufficiency of aortic valve	113	4.6 (1.1) *	17.1 (7.8) *	14333 (3401) *
Other endocardial cushion defect, ostium primum defect, unspecified	186	7.1 (1.0) *	15.1 (5.1) *	30284 (6591) *
Congenital mitral stenosis, congenital mitral regurgitation	75	4.4 (0.9) *	12.7 (8.4) *	13892 (4244)
Tetralogy of Fallot, infundibular pulmonic stenosis	291	4.8 (0.8) *	11.4 (4.0) *	21317 (3616) *
Hypoplastic left heart syndrome	160	3.3 (0.8)	9.5 (4.8) *	17433 (3510) *
Unspecified congenital anomaly of heart, other specified congenital anomaly of heart	641	3.7 (0.4) *	9.3 (2.6) *	16862 (1725) *
Pulmonary valve atresia, tricuspid atresia, pulmonary artery coarctation and atresia, other anomalies of pulmonary artery and pulmonary circulation	164	5.2 (0.7) *	8.1 (4.3) *	21389 (3688) *
Isolated pulmonary valve stenosis, congenital anomalies of pulmonary artery, other congenital anomalies of pulmonary valve, congenital pulmonary valve anomaly unspecified	172	4.7 (0.7) *	5.4 (3.7)	16372 (3643) *
Isolated unspecified defect of septal closure, ventricular septal defect	654	3.6 (0.2) *	5.3 (1.9) *	12950 (1287) *
Double outlet right ventricle, other bulbus cordis anomalies	102	4.9 (1.3) *	4.4 (4.3)	21092 (6705) *
Common truncus	18	6.1 (1.8) *	0	32164 (16986) *
**All CHD**	**4813**	**4.5 (0.2)** *	**12.3 (1.2)** *	**19650 (993)** *
**Without CHD**	**224,044**	**2.3 (0.02)**	**2.3 (0.1)**	**7939 (138)**

Individual diagnostic groups are ordered in descending order first based on the frequency of inpatient mortality and second by the frequency of mechanical ventilation. ASD, atrial septal defect; CHD, congenital heart disease; NE, not estimable due to no reported injury/poisoning hospitalizations; RSV, respiratory syncytial virus;RSVH, respiratory syncytial virus hospitalization.

^a^ No results are shown for diagnostic groups with ≤10 RSV hospitalization identified, consistent with Healthcare Cost and Utilization Project guidelines for National Inpatient Sample database analyses.

^b^ Geometric mean.

^c^ Adjusted to 2015 US dollars.

*P < .05 compared to the population without CHD.

**Table 4:** Percent Inpatient Mortality Among Children 12–23 Months of Age Hospitalized with RSV or UB, 1997–2013.

Condition^a^	Percent of Inpatient Mortality (SE)	
	RSV	UB
Transposition of great vessels	10.2 (5.3) *	0
Congestive heart failure	9.2 (3.7) *	2.5 (1.8) *
Cardiomyopathy	9.2 (5.1) *	0
Ebstein's anomaly	8.8 (8.4) *	0
Subaortic stenosis, congenital atresia and stenosis of aorta, congenital aortic stenosis, congenital obstructive anomalies	6.1 (4.2) *	0
Malposition of heart and cardiac apex with other anomalies of great vessels (heterotaxia), congenital anomalies of great veins	3.1 (3.1) *	3.4 (3.4) *
Congenital anomalies of aortic arch with other congenital anomalies of aorta, coarctation, interrupted aortic arch, congenital anomaly of aorta unspecified	2.6 (2.6) *	0
Isolated patent ductus arteriosus	2.4 (2.3) *	0
Isolated secundum atrial septal defect, partial anomalous pulmonary venous return	1.1 (0.8) *	0
**All CHD**	**1.5 (0.4)** *	**0.3 (0.2)** *
**Without CHD**	**0.1 (0.02)**	**0.02 (0.01)**

Individual diagnostic groups are ordered in descending order based on the frequency of inpatient mortality and secondarily the frequency of mechanical ventilation. Diagnostic groups are not listed if no mortality was observed with RSV or UB. ASD, atrial septal defect; CHD, congenital heart disease; RSV, respiratory syncytial virus; RSVH, respiratory syncytial virus hospitalization; UB, unspecified bronchiolitis.

^a^ No results are shown for diagnostic groups with ≤10 RSV hospitalization identified, consistent with Healthcare Cost and Utilization Project guidelines for National Inpatient Sample database analyses.

*P < .05 compared to the population without CHD

**Table 5:** UB Hospitalization Illness Severity Indicators for Children 12–23 Months of Age With CHD and Without CHD, 1997–2013.

Condition^a^	12–23 Months RSVH, N	Mean^b^ Days of Stay (SE)	Percent of Mechanical Ventilation Use (SE)	Mean^b^^,^^c^ Total $ Charge (SE)
Transposition of great vessels	71	3.5 (0.9) *	13.3 (9.0) *	17066 (5172) *
Congestive heart failure	304	4.1 (0.5) *	4.2 (2.4) *	16800 (2371) *
Cardiomyopathy	125	2.9 (0.9)	8.2 (5.3) *	12626 (2791) *
Ebstein's anomaly	50	2.1 (1.2)	0	10249 (2268) *
Subaortic stenosis, congenital atresia and stenosis of aorta, congenital aortic stenosis, congenital obstructive anomalies	94	3.7 (0.9) *	21.0 (9.4) *	18695 (5491) *
Malposition of heart and cardiac apex with other anomalies of great vessels (heterotaxia), congenital anomalies of great veins	146	2.8 (0.9)	27.4 (8.2) *	21086 (5169) *
Congenital anomalies of aortic arch with other congenital anomalies of aorta, coarctation, interrupted aortic arch, congenital anomaly of aorta unspecified	157	3.1 (0.7) *	12.0 (5.4) *	19525 (2883) *
Isolated patent ductus arteriosus	168	2.4 (0.7)	8.6 (4.8) *	12984 (2489) *
Isolated secundum atrial septal defect, partial anomalous pulmonary venous return	575	3.9 (0.5) *	11.0 (3.0) *	18055 (1919) *
Common ventricle, cor biloculare	22	4.5 (1.6) *	20.3 (18.2) *	20230 (13404)
Total anomalous pulmonary venous connection	29	5.0 (1.5) *	15.1 (14.4) *	22344 (9546) *
Congenital insufficiency of aortic valve	117	2.8 (0.4) *	0	13609 (2593) *
Other endocardial cushion defect, ostium primum defect, unspecified	116	3.6 (0.8) *	4.3 (4.3)	15032 (4425) *
Congenital mitral stenosis, congenital mitral regurgitation	74	3.3 (0.9) *	0	15368 (4840) *
Tetralogy of Fallot, infundibular pulmonic stenosis	269	3.3 (0.3) *	0	10322 (1368) *
Hypoplastic left heart syndrome	166	4.2 (0.6) *	5.7 (3.8) *	20261 (3922) *
Unspecified congenital anomaly of heart, other specified congenital anomaly of heart	601	2.7 (0.3) *	6.5 (2.1) *	12415 (1288) *
Pulmonary valve atresia, tricuspid atresia, pulmonary artery coarctation and atresia, other anomalies of pulmonary artery and pulmonary circulation	154	3.3 (0.9) *	14.4 (6.3) *	20654 (5574) *
Isolated pulmonary valve stenosis, congenital anomalies of pulmonary artery, other congenital anomalies of pulmonary valve, congenital pulmonary valve anomaly unspecified	157	2.1 (0.5)	3.2 (3.1)	10631 (1820) *
Isolated unspecified defect of septal closure, ventricular septal defect	405	2.3 (0.3) *	7.0 (2.6) *	10354 (1234) *
Double outlet right ventricle, other bulbus cordis anomalies	84	4.1 (1.1) *	22.5 (9.9) *	21143 (7852) *
Common truncus	38	3.6 (1.2) *	9.8 (8.4) *	13099 (5168)
**All CHD**	**3,754**	**3.0 (0.1)** *	**7.4 (0.9)** *	**13867 (629)** *
**Without CHD**	**190,276**	**1.7 (0.02)**	**1.1 (0.1)**	**6570 (98)**

To aid in comparison of results, individual diagnostic groups are ordered in descending order based on the frequency of RSV inpatient mortality and secondarily the frequency of RSV mechanical ventilation. CHD, congenital heart disease; NE, not estimable due to no reported injury/poisoning hospitalizations; RSVH, respiratory syncytial virus hospitalization; UB, unspecified bronchiolitis.

^a^ No results are shown for diagnostic groups with ≤10 RSV hospitalization identified, consistent with Healthcare Cost and Utilization Project guidelines for National Inpatient Sample analyses.

^b^ Geometric mean.

^c^ Adjusted to 2015 US dollars.

*P < .05 comparing to the population without CHD

**Fig 1:**
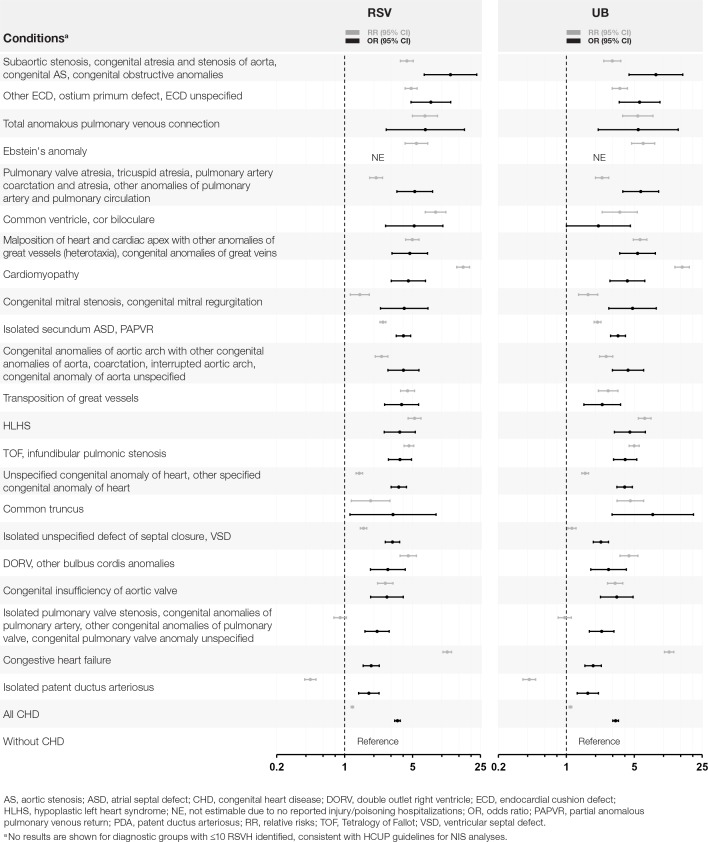
OR and RR of RSV or UB Hospitalization for Children 12–23 Months of Age With CHD by Diagnostic Group.

The OR of RSV hospitalization for children 12–23 months of age with CHD varied considerably across the diagnostic groups, which are presented in order of decreasing association in **Fig 1**. All CHD groups were associated with increased odds of RSV hospitalization, and the OR for all CHD diagnoses was 3.5. Fourteen groups had an OR of ≥3.5. No OR was estimable for Ebstein’s anomaly because there were no injury/poisoning hospitalizations reported. By diagnostic group, results by RR and OR were generally similar. The OR and RR of UB hospitalization by diagnostic group were generally similar to those observed with RSV (**Fig 1**).

When the intervals of 1997–2005 and 2006–2013 were analyzed separately, results were generally similar. A similar number of RSV hospitalizations among children with CHD occurred in both intervals (2361 and 2452, respectively). Among all RSV hospitalizations in children with CHD, the proportion with inpatient mortality was decreased (1.8% to 1.2% in 1997–2005 and 2006–2013, respectively), whereas, there were increases in the proportion with mechanical ventilation (10.1% to 14.5%), mean length of stay (4.3 to 4.8 days), and mean charges ($15,202 to $25,239 for CHD versus $6,777 to $9,392 for non-CHD, all in 2015 US dollars). The decline in RSV hospitalization mortality frequency was driven by a lack of reported mortality during 2006–2013 for aortic arch anomalies, heterotaxia, and isolated patent ductus arteriosus; RSV mortality frequency in other conditions was similar in both intervals. The OR of RSV hospitalization among all children with CHD was similar in both intervals, at 3.4 and 3.5, respectively. By individual CHD diagnostic groups, OR estimates declined among several higher-risk conditions, most notably aortic stenosis, congenital aortic arch anomalies, and pulmonary valve/artery anomalies. Similar trends were observed in UB, with the additional observation of an increase in the OR for UB hospitalization among all CHD children from 2.7 to 3.7.

## Discussion

Despite the previous observation that overall RSV hospitalization rates among children with CHD are lower at 12–23 months of age relative to children with CHD at 0–11 months of age [6], the present study demonstrates that compared to children without CHD, multiple specific CHD diagnoses are associated with substantial risks of mechanical ventilation and inpatient mortality as well as an increased risk of RSV hospitalization at 12–23 months of age with increased mean lengths of stay and total charges during RSV hospitalization. Five conditions (Ebstein’s anomaly, transposition of the great arteries, aortic stenosis, heterotaxia, and aortic arch anomalies) demonstrated a substantially elevated risk of RSV mortality, and 14 were associated with an elevated incidence of RSV hospitalization. The results from the present study provide clear evidence that for children with these high-risk types of CHD, RSV lower respiratory tract illness continues to be an important risk to their health through the second year of life. The results for RSV hospitalization are substantiated by the finding of similar results for UB hospitalizations and similar results when comparing 1997–2005 and 2006–2013. However, the lower severity of UB hospitalizations, as measured by length of stay, mechanical ventilation use, and inpatient mortality, highlights the more deleterious effects of RSV-specific disease in this population.

The current results are supported by the findings of Li et al. (2016), which compared patient characteristics and outcomes of children with hemodynamically significant CHD who received palivizumab and were enrolled in a large Canadian registry from 2005 to 2015.[27] Children receiving palivizumab in the second year of life represented a higher-risk subset of those receiving it in the first year, as only 28% of first year recipients continued for a second year and these infants had a much more complicated neonatal course. When outcomes were evaluated, the first and second year cohorts had similar hazards of RSV and overall respiratory-related illness hospitalizations, consistent with the present finding of significantly elevated risk among a high-risk subset of CHD patients in the second year of life.

The current analysis demonstrates that the previous observation of overall lower risk for all CHD patients in the second year of life is driven by the relatively low risk associated with the most prevalent conditions, such as isolated ventricular septal defect and patent ductus arteriosus. However, when the data are examined more closely, all of the diagnostic groups associated with a substantially increased risk of severe RSV disease represent hemodynamically significant CHD, including cyanotic heart disease, moderate-to-severe pulmonary hypertension, and acyanotic defects requiring medication or surgery—the same risk factors that identify those CHD patients most likely to benefit from immunoprophylaxis in the first year of life [15].

It should be noted that the impact of RSV on CHD patients hospitalized for RSV disease at 12–23 months of age is not merely confined to morbidity and mortality. In the more recent estimates from 2006–2013, there was a substantial increase in mean hospitalization charges for RSV in CHD ($25,239) versus non-CHD patients ($6,777), with even higher estimates among those with more severe CHD. This underlines the increased financial burden of RSV disease for CHD patients in the second year of life.

RSV immunoprophylaxis recommendations for children with CHD who are 12–23 months of age at the start of RSV season differ worldwide. Recommendations in Spain [28], the Netherlands [29], and Japan [30] support immunoprophylaxis for all children with hemodynamically significant CHD ≤24 months of age at RSV season start. Germany employs a risk-based approach, recommending immunoprophylaxis for those at 12–23 months of age with a “moderate risk” of RSV hospitalization [31]. Guidance in the United Kingdom [32] and Sweden [33] recommend immunoprophylaxis for children at 12–23 months of age with specific CHD diagnoses, such as CHD with pulmonary hypertension, congestive heart failure, or those with single ventricles. In contrast, guidance in Canada [34], Italy [35], and South Korea [8] does not recommend immunoprophylaxis for children with CHD at 12–23 months of age.

The observation for some CHD conditions of higher risks based on the RR approach versus the OR approach is likely due to under-diagnosis of some CHD conditions at birth. This was particularly pronounced for congestive heart failure and cardiomyopathy, both of which may be more frequently diagnosed following the birth hospitalization. Conversely, a higher risk by the OR versus RR may be due to over-diagnosis of CHD conditions at birth, perhaps due to ICD-9 coding of suspected but not yet confirmed diagnoses. In the present analysis, we found that double outlet right ventricle, endocardial cushion/ostium primum defects, Tetralogy of Fallot, and hypoplastic left heart syndrome commonly were diagnosed simultaneously among birth hospitalizations, consistent with the potential of over-diagnosis of some CHD conditions at birth before a precise diagnosis is established. ICD-9 coding should be more accurate at 12–23 months of age compared to the birth hospitalization [20, 36]. Additionally, the higher risk by OR could be due to less prevalent use of CHD codes among children after surgical correction or clinical resolution of their disease. The elevated risks by the case-control approach for these diagnostic groups are consistent with a significantly elevated risk among children with continuing CHD pathology.

The case-control analysis relies on the assumption that CHD codes are equally prevalent among hospitalizations for RSV or UB relative to those for injuries and poisonings. Differential use of CHD codes for RSV or UB versus injuries and poisoning would skew the OR estimates. However, the high prevalence of less severe CHD codes (eg, isolated patent ductus arteriosus) reported for children 12–23 months of age hospitalized with injuries and poisonings suggests that reporting of CHD codes in children with a history of CHD is commonplace, even for non-cardiopulmonary hospitalizations. Additionally, the incidence of injuries and poisonings among children 12–23 months of age with CHD could be decreased due to reduced activity or increased due to increased risks of metabolic bone disease [37], physical abuse [38], or drug-related adverse events [39]. The decreased or increased incidence of injury/poisoning among children with CHD would lead to an overestimate and underestimate of the OR for RSV hospitalization, respectively. However, the finding that the specific injury/poisoning ICD-9 codes employed in the analysis occurred at a similar proportional frequency among CHD and non-CHD children supports the validity of the OR analysis. Further, collectively the above factors did not appear to have a significant impact on the results, given that the RR and OR estimates of risk are similar in many diagnostic groups and the overall OR for all CHD at 12–23 months of age is similar to the previous observation by Boyce et al. [6].

Strengths of the current analysis relate to use of 17 years of data from a large, nationally representative database of US hospitalizations. This large sample size enabled a robust examination of RSV and UB hospitalization illness severity and risk by individual CHD diagnostic groups, many of which are extremely rare. Additionally, the estimation of hospitalization risks using both cohort and case-control designs helped to address limitations inherent in both approaches. The fact that similar results were observed using the two methods for most conditions supports the validity of the current findings.

Limitations of the current study include the following: Data from the NIS database do not enable identification of which patients had received RSV immunoprophylaxis. Selective use of RSV immunoprophylaxis during many of the years studied may have reduced the overall incidence of RSV hospitalization among the CHD population [40, 41], although it is likely that not all high risk children 12–23 months of age with CHD received immunoprophylaxis [42]. Similarly, no patient-level data were available on the status of surgical correction for the hospitalized children. This would be expected to influence the risk profile of certain patient subgroups because surgically repaired patients should no longer be at high risk once recovered from surgery. Therefore, these analyses and conclusions are conservative, with the current results representing the risk associated with all infants with a diagnostic code of the condition, including those with and without surgical correction. Not all cardiac surgery for CHD is performed in the first year of life. The Agency for Healthcare Research and Quality has documented the age at which major CHD surgeries are performed in US children, demonstrating that 23% occur during the neonatal period, 38% from 1–11 months of age, 14% from 12–23 months of age, 9% from 3–5 years of age, 10% from 6–12 years of age, and 6% from 13–17 years of age [43]. Therefore, the finding that 39% of major pediatric surgeries for CHD are conducted after 11 months of age would suggest that some children with severe CHD continue to have pathophysiology that would place them at increased risk for severe RSV disease at 12–23 months of age.

## Conclusions

Using data from a nationally representative database from a period of 17 years, this study found that certain CHD diagnoses place children at an elevated risk of RSV hospitalization at 12–23 months of age with considerable hospital morbidity and mortality. The highest-risk CHD diagnoses fit the definition of hemodynamically significant CHD, and such patients would benefit from all efforts to prevent the incidence of severe RSV disease in the second year of life.

## Supporting information

S1 Table Coincidence of CHD Diagnostic Categories Among Live Births with CHD Diagnoses, 1997–2013. (XLSX)

S2 Table Coincidence of CHD Diagnostic Categories Among Children 12–23 Months with CHD Diagnoses, 1997–2013. (XLSX)
